# Brain Deactivation in the Outperformance in Bimodal Tasks: An fMRI Study

**DOI:** 10.1371/journal.pone.0077408

**Published:** 2013-10-14

**Authors:** Tzu-Ching Chiang, Keng-Chen Liang, Jyh-Horng Chen, Chao-Hsien Hsieh, Yun-An Huang

**Affiliations:** 1 Department of Psychology, National Chung Cheng University, Min-Hsiung Township, Chia-Yi County, Taiwan; 2 Department of Psychology, Ohio State University, Columbus, Ohio, United States of America; 3 Department of Psychology, National Taiwan University, Taipei, Taiwan; 4 Electrical Engineering, Interdisciplinary MRI Laboratory, National Taiwan University, Taipei, Taiwan,; University of Leuven, Belgium

## Abstract

While it is known that some individuals can effectively perform two tasks simultaneously, other individuals cannot. How the brain deals with performing simultaneous tasks remains unclear. In the present study, we aimed to assess which brain areas corresponded to various phenomena in task performance. Nineteen subjects were requested to sequentially perform three blocks of tasks, including two unimodal tasks and one bimodal task. The unimodal tasks measured either visual feature binding or auditory pitch comparison, while the bimodal task required performance of the two tasks simultaneously. The functional magnetic resonance imaging (fMRI) results are compatible with previous studies showing that distinct brain areas, such as the visual cortices, frontal eye field (FEF), lateral parietal lobe (BA7), and medial and inferior frontal lobe, are involved in processing of visual unimodal tasks. In addition, the temporal lobes and Brodmann area 43 (BA43) were involved in processing of auditory unimodal tasks. These results lend support to concepts of modality-specific attention. Compared to the unimodal tasks, bimodal tasks required activation of additional brain areas. Furthermore, while deactivated brain areas were related to good performance in the bimodal task, these areas were not deactivated where the subject performed well in only one of the two simultaneous tasks. These results indicate that efficient information processing does not require some brain areas to be overly active; rather, the specific brain areas need to be relatively deactivated to remain alert and perform well on two tasks simultaneously. Meanwhile, it can also offer a neural basis for biofeedback in training courses, such as courses in how to perform multiple tasks simultaneously.

## Introduction

 While some people excel at performing two tasks simultaneously, others can only effectively perform tasks sequentially. Past research has demonstrated that the performance of multiple tasks is not hindered when both tasks are executed by different modalities, such as tasks involving vision and audition [1-3]. These studies suggested the presence of multiple processors for each modality [4]. This leads to the question of whether the source of processors for the different modalities is the same or whether the processing resource is modality specific. Some studies have suggested that each modality contains its own processing resources (i.e., that the resources are modality specific) which, to some extent, are independent from those of other modalities [5-13]. On the other hand, the observation that people cannot perform well on bimodal tasks is also supported by the evidence that processing resources are shared among modalities (i.e., modality independent), and therefore, performance of bimodal/dual tasks depends on interactions between the processing for each of the tasks [13-18].

 The processing dependency in the two mechanisms involved in bimodal divided attentional tasks can be elucidated through the different predictions in our imaging study. When two tasks with separated streams of modality processing are adopted, such as a visual “what” task and a auditory “where” task [19-21], which have been suggested to have little cross-modal interaction [22], the predictions for the two mechanisms will be different. If modality specific processing exists, distinct brain areas, including stimuli-related processors, should be found for each unimodal task. On the other hand, if modality independent processing exists, then shared and common brain areas should be found for each unimodal task. Although the current research paradigm in the literature is to manipulate the task demands of one modality to examine the effect on the other [16,23-25], the manipulation of task demands may risk exceeding the limit of one modality’s processing resources, causing its interaction with the other modality. It may become a confounding variable to differentiate the two concepts, because total processing resources are limited regardless of whether they are modality specific or modality independent. As a result, the present study did not aim to manipulate the task demands of modalities. 

 Although past research showed activation, for the divided attention tasks, in the prefrontal areas [26-28], anterior cingulate cortex [27], and the inferior parietal lobes [28], little agreement has been reached on which brain areas are required to outperform in bimodal tasks, especially compared to those who performed well on only one of the bimodal tasks. Therefore, the present study aims to answer the question by using functional magnetic resonance imaging (fMRI). The study is important not only to determine the possibility of deciphering the neural basis of one intriguing cognitive process of human minds, but also because it shows applicability to educational training, such as instruction in how to excel at two tasks simultaneously.

## Methods

### Subjects

Twenty-four healthy subjects (11 males and 13 females) between 19 and 28 years of age (mean 21.8, SD 2.4) participated in this study. All subjects had normal or corrected to normal vision and were right-handed. Written informed consent was obtained from all subjects in accordance with the Declaration of Helsinki and ethical consent for performing the fMRI study was granted by the Ethics Committee of the Department of Psychology, National Chung Cheng University, Taiwan (ethical code: 098031604). 

### Experimental setup and stimuli

**Figure 1:**
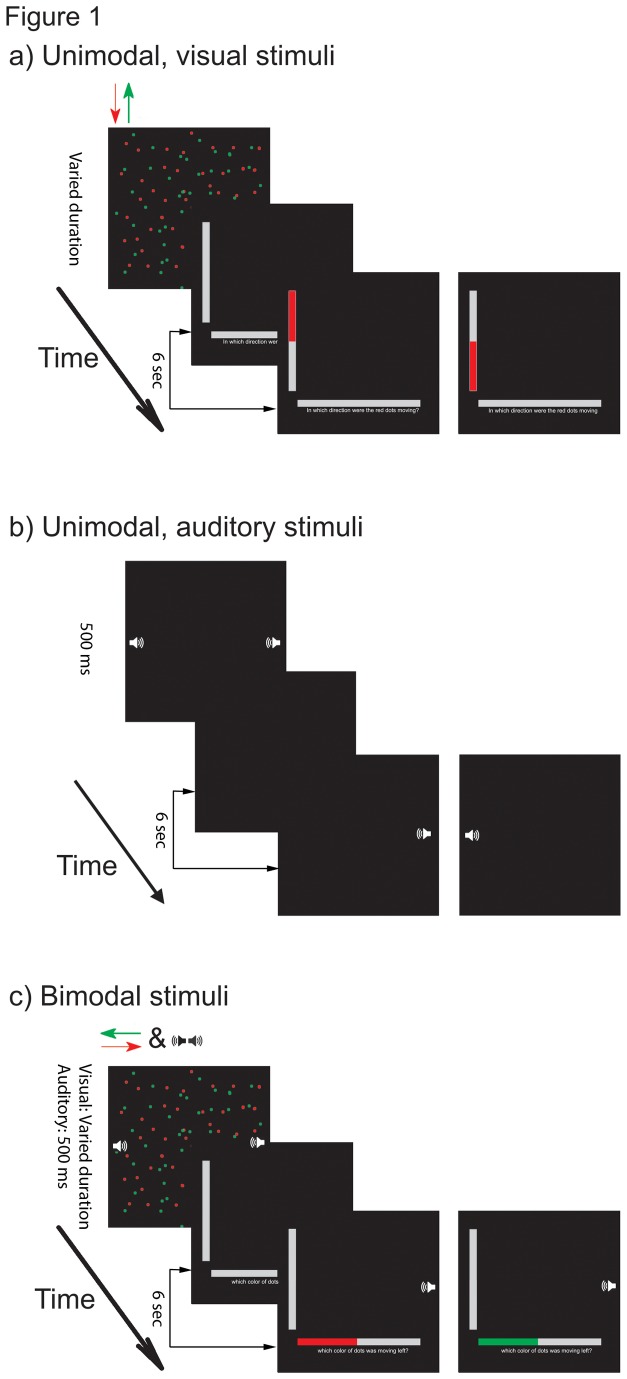
Schematic diagram depicting the study task. a) The unimodal visual stimuli consisting of red and green dots moving either vertically or horizontally were presented for a specific duration. A question then appeared to which the subject responded using the keypad on the right hand and the response immediately showed on the relevant grey bar. The examples shown here are questions related to the moving direction of the red dots. b) The unimodal auditory stimuli involved the presentation of two different pitches on the left and right ear for 500 ms and icons were shown on the screen. After the presentation, a black screen appeared to which the subject responded using the keypad on the left hand in order to indicate which ear was presented with the lower frequency. The response immediately showed on the icon at the relevant location. c) The bimodal task presented the visual and auditory stimuli at the same time. After presentation of the stimuli, the subject needed to respond to the subsequent visual question with the keypad on the right hand and indicate the lower frequency with the keypad on the left hand. The sequences of visual and auditory responses were irrelevant. In the examples illustrated here, the subjects indicated that the right ear heard the lower frequency of tones and the red or green dots were moving to the left.

The experiment contained three tasks, including visual, auditory and bimodal tasks (Figure 1). The visual task was adopted from Chiang et al. [29], consisting of either 50 green and 50 red dots or 50 yellow and 50 blue dots on a black background. Half of the dots of each color moved in opposite directions along either a horizontal or vertical axis at a speed of 4.30°/s. These dots moved at variable angles within a range of 0.14° of visual angle perpendicular to the original direction of movement. The other half of the dots of each color flashed at random locations in order to increase the task difficulty. Before testing, equiluminance was separately established for each subject by flicker photometry [30]. The task required subjects to identify which color was moving and in what direction by using a right-hand keypad to answer one of two questions that randomly appeared after the stimulus presentation. One question asked, “Which color of dots was moving *direction*?” (The word *direction* was replaced by *up, down, left or right*, as appropriate.) The other question asked, “In which direction were the *color* dots moving?” (The word *color* was here replaced by *green, red, yellow* or *blue*, as appropriate.) Furthermore, the questions were relevant to the stimuli. For instance, if the subjects were shown red and green dots, they were not asked about the direction of blue or yellow dots, only that of red or green dots. If they were shown dots moving horizontally, they were not asked about the color of dots moving vertically. The combination of stimuli and related questions were balanced in a random sequence such that each type of stimulus was followed by each of the possible questions in turn. Each subject’s response to the visual stimuli was categorized as either correct or incorrect based on its objectively assessed accuracy. 

 The auditory task consisted of two pure tones, each randomly presented to one ear for 500 ms. One tone was fixed at the piano key f4 (349.2 Hz), while the other was randomly selected from between the piano keys g4 (392.0 Hz) and b6 (1046.5 Hz). The intensity of the two tones was adjusted to be subjectively equal in loudness for individual subjects (see procedure section below for details). The subject was then instructed to indicate which ear received a lower pitch by pressing a left-hand keypad. The response was categorized as either correct or incorrect based on its objectively assessed accuracy. 

 In the bimodal divided attentional task, the visual and auditory stimuli were presented at the same time to subjects. The subject was instructed to complete both tasks as accurately as possible, regardless of the order of response. The visual stimuli were constructed using the Cogent Graphics toolbox (available at www.vislab.ucl.ac.uk) and the auditory stimuli were constructed using MATLAB (MathWorks Inc.). Both sets of stimuli were executed in MATLAB on a PC computer and presented to functional magnetic resonance imaging (fMRI) compatible goggles (14.25° x 10.71° of visual angles in width and height, VisuaStimXGA, Resonance Technology Inc., Northridge, California, USA).

### fMRI scanning methods

All functional scanning was performed in a 3T Bruker 30/90 Medspec fMRI scanner fitted with a standard birdcage head coil (BrukerBioSpin MRI GmbH, Ettlingen, Germany). An echo planar imaging (EPI) sequence was applied for functional scans, measuring blood oxygen level dependent (BOLD) signals (echo time, TE = 30 ms, repeat time, TR = 3s). Each brain image was acquired in an interleaved sequence from the bottom of the brain to the top comprising 35 axial slices, each of which was 3.75 mm thick with no gap between the slices. Images were acquired at a resolution of 3.75 × 3.75 × 3.75 mm and covered nearly the entire brain. The first seven volumes of each scanning session were discarded to allow for T1 equilibrium effects. T1 weighted, axial anatomical scanning was performed after functional scanning to obtain high-resolution structural images with the same scanning sequence as that of EPI. Each volume comprised 35 axial slices, at a resolution of 0.9375 × 0.9375 × 3.75 mm (TE = 39.4 ms, TR = 614.2 ms, flip angle 90, field of view [FOV] = 240 mm).

### Procedure

Pretests were first conducted with the subjects outside the scanner in order to gather appropriate parameters for the auditory and visual tasks, such as loudness adjustment and the duration of visual stimuli. The loudness of auditory stimuli was adjusted using a method in which the subject was first presented with two pure tones, each in on one ear randomly. One of the two pure tones was always fixed as the piano key f4 (349.2 Hz). The other tone was randomly selected among the piano keys from g4 (392.0 Hz) to b6 (1046.5 Hz). The subject adjusted the loudness of the tone to match that of f4 by decreasing or increasing the volume of the tone. Once the subject felt that the loudness of the two tones sounded equal, the same method was then applied to the next randomly selected tone between g4 and b6. After completing the adjustment of all of the tones, the subjects were tested behaviorally in 32 trials in which each trial involved random presentation of a tone from g4 to b6 and a fixed tone, f4, to both ears. The arrangement of tones and ears (i.e., which tone was presented to which ear) was randomly assigned by each trial but the total number of tones f4 presented to the right ear was 16 by the end of the trials. If the subject failed to discriminate with 68% accuracy which ear received the tone with the lower pitch, then the loudness adjustment procedure would start again. If the subject still failed the accuracy criterion, then he or she would not be qualified to continue participating in the experiment. The 68% accuracy requirement was based on the statistical reasoning that the minimum probability of obtaining a value significantly higher than 50% is 0.6732 when the α value is set to 0.05.

 Next, the duration of visual stimulus presentation for each subject was determined using the method of limits. The subject was first provided with the longest duration of stimulus presentation with which to perform feature binding, namely two seconds. This was to ensure that the subject could respond correctly in five consecutive trials within this duration. The subject was then provided with successively decreasing durations of stimulus presentation with each duration reduced by half until the subject reported that he or she could no longer respond correctly in three out of the last five consecutive trials. Next, the subject was provided with the shortest duration of stimulus presentation with which to perform feature binding, namely 32 ms, to ensure that the subject could not respond correctly in five consecutive trials within this duration. The subject was then provided with increasing durations of stimulus presentation, each successively increasing by twofold until the subject reported that he or she could respond correctly in four out of five consecutive trials. The descending and ascending order of duration was repeated three times per subject, while varying the longest and shortest durations. After completion of this process, a constant duration was chosen and tested for one block of 32 trials to ensure that the subject could identify the visual stimulus correctly in at least 68% of the presentations. If a subject failed to meet the performance criteria, the stimulus duration was changed and testing was repeated. If the subject still failed the accuracy criterion, then he or she would not be qualified to continue participating in the experiment. The optimal visual stimulus duration among the subjects ranged from 66.7 ms to 800.16 ms (mean = 199.16 ms, SD = 179.8 ms).

 After the stimulus parameters were appropriately chosen, the subject was sent into the fMRI scanner to start formal experiments. The fMRI scanning consisted of four sessions of 40 trials including eight randomly included control stimuli. The first session consisted of a visual unimodal task and the second session was an auditory unimodal task. The third and fourth sessions were bimodal tasks. In each trial of a visual unimodal task, the visual stimulus was presented alone for a chosen duration and the headphones were silent. The visual stimulus subsequently disappeared and a question appeared at the bottom of the screen for a six-second period (equals two times of TR = 3 sec), during which the subject indicated his or her response with a right-hand keypad. The next trial automatically began after the six seconds had passed. The control stimulus for the visual task was a white cross presented at the center of the screen for the same duration as the visual stimuli, followed by grey bars without questions. Subjects did not need to respond to the control stimulus. The second session was the auditory unimodal task, beginning with the presentation of a black screen (i.e., no visual stimuli) and two tones simultaneously, one in each ear, for 500 ms, as well as two white speaker icons on the left and right side of the screen. A black screen was then presented for six seconds and the subject indicated with a left-hand keypad which ear received the tone with the lower pitch. A corresponding speaker icon was shown on the screen when subjects chose their responses. The next trial automatically began after six seconds. The control stimulus for the auditory task was white noise presented to both ears with the same loudness as the f4 tone.

 The third and fourth sessions of fMRI scanning each consisted of a bimodal task. These tasks presented visual and auditory stimuli together. After that, the visual question appeared on the screen for a six-second period during which the subjects indicated their responses to both visual and auditory questions by pressing the right- and left-hand keypad, respectively. The next trial automatically began after six seconds had passed. The control stimulus for the bimodal task was a combination of visual and auditory control tasks, i.e., a white cross at the center and white noise to both ears. 

### fMRI data analysis

The fMRI data were analyzed only when the behavioral data met the criteria of accurate performance on more than 68% of the unimodal tasks. Those subjects whose behavioral data did not meet the criteria for either visual or auditory tasks were excluded from the study (5 out of the 24 subjects). Good performance in this study meant subjects’ performance was over 68% in accuracy. In the unimodal tasks, good performance formed a kind of baseline which allowed us to examine the change in performance during the bimodal task. 

 The fMRI data were initially preprocessed using SPM8 software (Wellcome Department of Imaging Neuroscience, London, UK, http://www.fil.ion.ucl.ac.uk/spm) in which each image was first realigned to the average of all volumes obtained during the fMRI scanning and coregistered to each subject’s structural image. The images were then spatially normalized to the canonical template provided by SPM8 software. In addition, the images were spatially smoothed with a Gaussian kernel of 8 mm full width at half maximum (FWHM). The preprocessed data were then subjected to first level analysis using a voxelwise general linear model (GLM). The design matrix of the first level analysis consisted of four sessions of regressors. The first session consisted of the onset time of correct, incorrect and control trials of the visual unimodal task. The second session consisted of the onset time of correct, incorrect and control trials of the auditory unimodal task. The third and fourth sessions consisted of five regressors for the onset time of the combined performance on the dual tasks, including the trials with correct responses to visual and auditory tasks, the trials with correct visual and incorrect auditory responses, the trials with correct auditory and incorrect visual responses, the trials with incorrect responses on both tasks, and the control trials. Each session also contained motion correction parameters which were treated as effects of noninterest. Appropriate regressors were convolved with the default SPM hemodynamic response function (HRF) with two additional derivatives of time and spatial dispersion. 

 Restricted maximum likelihood (ReML) inference was used to estimate the parameters of the model. The estimated parameters were applied to various contrasts of interest that were then applied to second level analysis (i.e., between subjects). We used a one factor analysis of variance (ANOVA) at three levels (i.e., canonical, time derivative and spatial dispersion) with classical inference (ReML) estimation. The final outcome of these analyses was a group analysis resulting from the pooling of data across subjects. The statistical results were based on the uncorrected p value (p = 0.001) and used to choose clusters (voxel number ≥ 4 in cluster) that passed the criterion of multiple comparisons with a familywise error (FWE) corrected p value of 0.05 at the cluster level. The coordinates of all activation sites were based on the reference brain atlas provided by the Montreal Neurological Institute. The MarsBaR toolbox (http://sourceforge.net/projects/marsbar/files/) was used to create masks of the visual and auditory tasks as well as the region of interest (ROI) analysis of contrasts in the bimodal tasks. The selection of ROIs were based on the functional contrasts of unimodal and bimodal tasks. The imaging results were illustrated with the xjView toolbox (http://www.alivelearn.net/xjview). 

## Results

Table 1 shows a summary of behavioral results for performance on the unimodal and bimodal tasks. The mean accuracy for unimodal tasks was significantly higher than chance level (t_18_ = 13.54 and 13.87 for visual and auditory tasks respectively, p <0.001). Variations in duration did not affect performance because the accuracy was kept at the same level among subjects. The results were used as a baseline for the subjects who could perform single tasks well. During the bimodal tasks, each subject had two sessions of the bimodal task, which resulted in 36 sessions (degrees of freedom) for analysis. The result of each session was viewed as an independent subject because subjects’ performance in the two sessions were not identical (see Table 2). For instance, some subjects performed well in one modality in one session but performed well or badly in both modalities in the other session. Another example is that some subjects performed well in one modality in the first session and in the other modality in the next session. The results presented here were based on the performance in each session. Some subjects performed well in one modality in at least one session - eight subjects for ‘visual good’ and nine for ‘auditory good’; e.g., mean 10.1 trials for visual-good responses, SD = 2.6, accuracy = 0.73, SD = 0.07, t_7_ = 13.54, p < 0.001, compared to 0.5 in accuracy) - but poorly in another; e.g., mean 4.7 trials for auditory-bad responses, SD = 2.4, accuracy = 0.52, SD = 0.07, t_7_ = 0.92, p = 0.3896. In contrast, 13 participants in the bimodal task, in at least one session, performed well in both visual and auditory modalities (mean trial number = 17.4, SD = 1.6, accuracy = 0.71, SD = 0.08, t_14_ = 13.54, p < 0.001, compared to 0.5 in accuracy). 

**Table 1:** The mean accuracy and the average trial numbers of task performance.

Tasks		Mean ACC (SD)		Mean Trial Numbers (SD)	
		Visual	Auditory	Visual	Auditory
Visual task		0.7432* (0.0762)	--	32 (--)	--
Auditory task		--	0.7284* (0.0699)	--	32 (--)
Bimodal tasks	Both good	0.7101* (0.0503)	0.7467* (0.0740)	17.4 (1.6)	17.4 (1.6)
	Visual good	0.7275* (0.0748)	0.5225 (0.0694)	10.1 (2.6)	4.7 (2.4)
	Auditory good	0.5533 (0.0444)	0.7667* (0.0620)	11.3 (1.8)	4.6 (1.7)

The subject number of ‘Both good’, ‘Visual good‘ and ‘Auditory good‘ in the bimodal task was 15, 8 and 9, respectively. ‘*’ indicates statistical significance, p < 0.001, compared to 0.5. ACC = accuracy. SD = standard deviation of mean accuracy or trial numbers. ‘--’ indicates not applicable to the joint conditions.

**Table 2:** Subjects’ performance in bimodal tasks.

Subject #	Good Performance in One Modality		Good Performance in Both Modalities
	Visual	Auditory	
1		x	x
2	x		x
3	x		
4		x	x
5	x		x
6		x	x
7	x		x
8			x
9		x	x
10		x	
11			xx
12	x	x	
13	x	x	
14		x	x
15		x	x
16	x		
17	x		
18			xx
19			xx

Subjects perform two sessions in the experiment and as a result, each row has at most two “x”. Each “x” indicates what good performance was done by subjects in one session, either in one modality (visual or auditory) or in both modalities. “xx” indicates the good performance in both sessions. Some subjects have only one “x” because their performance in one session reaches the criteria of good performance (see details in the Methods) but fails in the other session.

**Figure 2:**
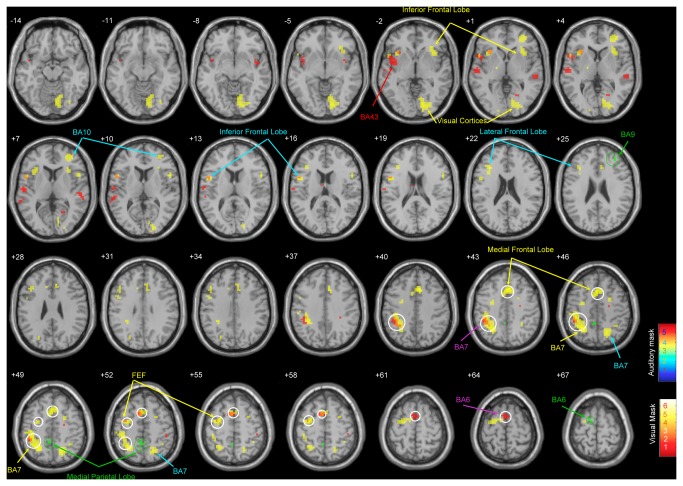
Schematic results of the statistical parametric mapping (SPM) analysis of the contrasts. An uncorrected threshold of p = 0.001 was used for the voxel illustration. The SPM results were superimposed onto the SPM toolbox, xjView, with a template of brain structure indexed for the coordinate of the z axis according to the MNI space. Unimodal stimuli versus rests were indicated in yellow and red words for visual and auditory activation, respectively (FWE p< 0.05).Brain regions activated by visual and auditory stimuli, selected with FWE p value between 0.05 and 0.08, were in cyan and purple words, respectively, and were also depicted in yellow and red colors in the corresponding brain areas. A mask was formed to examine the bimodal task versus the average of unimodal tasks. The results shown in the green words and circles indicate three activated areas including the medial parietal lobe, BA9 and BA6, all outside the mask (FWE p value < 0.05). Finally, fifteen areas formed a ROI to examine the performance of subjects that outperformed bimodal tasks versus those who performed well in only one of the bimodal tasks. The ROI analysis shown in white circles revealed that the medial frontal lobe, FEF, BA6, BA7, and medial parietal lobe were negatively correlated with the contrast (t test, p < 0.05).

 Imaging results are shown in Table 3 for detailed statistics and Figure 2 for illustration. Compared with the control condition, the visual unimodal tasks (including both the correct and incorrect trials) resulted in activation of the visual cortices, the lateral parietal lobe, the medial frontal lobe, frontal eye field (FEF), and inferior frontal lobe (all p < 0.05 with FWE correction). Imaging results of the auditory unimodal task (including both the correct and incorrect trials), compared to the control conditions, showed a significantly activated auditory cortex including BA43 compared to the rest condition (p < 0.05 with FWE correction). 

**Table 3:** Brain areas identified by the results of imaging contrasts.

Contrasts	Brain Areas	Voxel # in Cluster of Effect Size (d)	Peak MMI Coordinate			t Value	p Value
			x	y	z		
Visual task vs Control	Visual cortices	157	12	-82	-5	6.13	0.00002
	Lateral parietal lobe/BA7	181	-29	-52	48	6.74	0.000008
		32	20	-63	48	5.36	0.07
	Medial frontal lobe	91	4	23	44	6.80	0.001
	FEF	70	-29	-3	55	5.77	0.004
	Inferior frontal lobe	37	34	19	2	4.81	0.04
		30	-48	4	18	5.42	0.08
	Lateral frontal cortex	31	-33	27	21	6.32	0.08
	BA10	30	34	49	6	4.95	0.08
Auditory task vs Control	BA43	44	-40	0	-1	5.05	0.029
	Lateral parietal lobe/BA7	34	-44	-37	44	5.35	0.06
	BA6	35	-7	12	51	5.84	0.06
Bimodal vs Unimodal tasks	BA6	8	-14	0	66	4.48	0.003
	Medial parietal lobe	20	-3	-44	51	4.34	0.02
	BA9	4	34	46	25	4.06	0.02
Both good vs One good in bimodal task (ROI analysis)	Medial parietal lobe	d = -4.73	-3	-44	51	-1.76	0.04
	BA7	d = -4.72	-44	-37	44	-1.74	0.04
	BA6	d = -7.95	-7	12	51	-2.53	0.008
	FEF	d = -3.72	-29	-3	55	-1.74	0.04
	Medial prefrontal lobe	d = -8.24	4	23	44	-2.02	0.02

Four contrasts were conducted including visual stimuli vs. control, auditory stimuli only vs. control, the bimodal tasks vs. the average of unimodal tasks, and good performance in both bimodal tasks vs. good performance of either one of the bimodal tasks. The first three contrasts were used at the second level of SPM with a FWE of p < 0.05 at the cluster levels. The fourth contrasts were assessed using an ROI analysis with the *t* test. Positive *t* values indicate activation of contrasts and negative *t* values indicate deactivation of the contrast. BA6 = Brodmann area 6, BA7 = Brodmann area 7, BA9 = Brodmann area 9, BA10 = Brodmann area 10, BA43 = Brodmann area 43, BA47 = Brodmann area 47, FEF = Frontal eye field, MNI = Montreal Neurological Institute.

 In order to examine whether additional brain areas were recruited during performance of the bimodal task compared to the average of the unimodal tasks, a group of 10 brain areas (12 clusters) were formed as a mask (i.e., the visual and auditory tasks in Table 3). The 10 brain areas were selected from the FWE corrected p value < 0.08 in which the six mentioned brain areas mentioned above were also included. The reason we chose voxels of p < 0.08 to form the mask was to increase the statistical power (i.e., 1 - ®) and to avoid missing some voxels that truly belonged to the processing sites of visual and auditory information. Imaging results contrasting the bimodal task (including both the correct and incorrect trials) with the average of the single tasks revealed three additional brain areas outside the mask, including BA6, the medial parietal lobe and the dorsolateral prefrontal cortex (BA9), that were necessary to perform the bimodal task (all p < 0.05 with FWE correction, see Table 3 and green letters and circles in Figure 2). Inside the mask, there were no significant results of the contrast. 

 Furthermore, the 10 brain areas found in the unimodal tasks, along with the three additional areas defined by the contrast of bimodal versus unimodal tasks were used to run ROI analysis of the contrast between good performance in both tasks and good performance in only one task in the bimodal task. The sizes of ROIs were listed in the voxel numbers in Table 3. This helped us assess whether the behavioral differences can be accounted for by the differential activity of these ROIs. Behaviorally good performance in both modalities in the bimodal task resulted in relative deactivation of some brain areas such as BA6 (t_17_ = -2.53, p < 0.05), FEF (t_17_ = -1.74, p < 0.05), medial frontal area (t_17_ = -2.02, p < 0.05), medial parietal lobe (t_17_ = -1.76, p < 0.05), and BA7 (t_17_ = -1.74, p < 0.05; see Table 3 and white circles of Figure 2). 

## Discussion

 The results of our study showed that subjects who performed well on the bimodal tasks showed decreased brain activity in some visual, auditory and parietal lobes, compared to those who could only perform well on one of the two simultaneous tasks. Meanwhile, when subjects performed a bimodal task, extra brain areas such as BA6, the medial parietal lobe and the dorsolateral prefrontal cortex were required, compared with the unimodal single tasks. Our results showing the activation of distinct brain areas for unrelated visual and auditory tasks support the concept of modal specific information processing. 

 The visual and auditory tasks in our experiment aimed to study the distribution of processing among different modalities. Unlike the cross-model integration paradigm discussed by Spence and Driver [31], in which cues were manipulated to influence judgment in tasks, our present study does not manipulate cues before presentation of stimuli. Hence, the characteristics of the visual and auditory tasks can be viewed as independent. Alais et al. [14] found that the individual thresholds of the bimodal stimuli (pitch discrimination and visual contrast tasks) were not affected by each other, suggesting that people have the ability to perform two concurrent tasks well. This differs in part from our results, in which some subjects could outperform in the bimodal task but others could not. The discrepancy in our results and those of Alais et al. [14] may be attributed to the different criteria of task measurement. Our task adopted a criterion of approximately 70% accuracy as the index of good performance, whereas Alais et al. [14] measured the threshold (i.e., 50% accuracy) of visual and auditory modalities. Unlike a 70% accuracy requirement, an unchanged threshold in the combination of visual and auditory tasks does not mean the performance of the two tasks will always be good. 

 Our results showed that distinct brain areas are recruited to perform visual and auditory unimodal tasks, indicating that each modality may have its own modal specific information processing. These results are compatible with previous studies [1-3,5,6,8,10,13]. Furthermore, our results showed that additional brain areas are required for performance of bimodal tasks compared to the summation of brain areas activated in unimodal tasks. This is supported by behavioral data demonstrating that performance of bimodal tasks requires more than the simple addition of two unimodal stimuli [32-34], as well as by the physiological evidence of dorsolateral prefrontal cortices involved in bimodal tasks [26-28]. Although the current study did not directly manipulate attention, our results still reveal reliable results that match with past research about the allocation of attentional resources. For example, the medial parts of the parietal lobe have been found to be domain independent and associated with shifts in attention [26-28,35]. Furthermore, BA9 and BA6 have traditionally been considered to be related to decision-making that involves the selection of a course of action based on immediate or past information from trials [36-38]. In a recent model of “hierarchical control over decision making”[39], BA9 and BA6 were responsible for perceiving “episodic” and “sensorimotor” information, respectively, and working together to select the appropriate actions. Collectively, the evidence suggests that the additional brain areas recruited for performance of bimodal tasks may play a role in the processing of information, a role that is compatible with the concept that processing of bimodal stimuli is more efficient than that of unimodal stimuli [34,40,41]. 

 The result that subjects who performed well in the bimodal task displayed relative deactivation in areas such as medial frontal areas, FEF, BA7, and the medial parietal lobe, compared to subjects who performed well in only one of the two simultaneous tasks, suggests that the brain needs to be deactivated to remain alert, in order to outperform two tasks simultaneously. These brain areas found in the study match the brain areas of the default network or default mode of the brain [42,43]. Although the brain only weighs 2% of total body weight, it consumes 20% of the body’s energy. As a result, the brain’s activities can be recorded even in a rest state. The primary brain areas of the default network include the dorsolateral and ventral medial frontal cortex, the posterior cingulate cortex (especially the retrosplenial cortex), the inferior parietal lobe, the lateral temporal cortex, and the hippocampal formation [44]. One of the main features of the default network is that while subjects are performing relevant goal directed tasks, the deactivated brain areas can be measured, resulting in a negative correlation between the corresponding brain areas and the tasks [45]. The default network has been considered to be related to internal cognitive activities such as episodic memory and attention shifts towards the internal state [46]. When task difficulty increases, the deactivation of the default network is linearly increased [47]. This implies that when subjects efficiently and simultaneously handle multiple tasks, they may pay attention to the information entering the brain and/or resources allocated to different modalities, rather than paying attention to external information. These findings were also supported by Tomasi et al. [48] and Esposito et al. [49] in their study suggesting that reallocation of attentional resources ensures active involvement in the ongoing tasks.

 In summary, the results of the present study suggest that the perceptual processing of vision and hearing are modal specific, rather than shared or overlapping. However, the total amount of resources is limited. When bimodal tasks are executed, compared to unimodal tasks, activation of extra brain areas outside of the processing of vision and hearing are required, suggesting that interaction between the modalities is necessary to cope with the demand of bimodal tasks. The most important finding is that relatively decreased activity of some visual, auditory and prefrontal brain areas was found for those subjects that performed well in bimodal tasks compared to those who performed well in only one of the bimodal tasks. This suggests that the shift to internal states would also be necessary to handle the demand of performing concurrent bimodal tasks. Bimodal tasks cannot be like unimodal tasks in requiring as many resources as they want, because that results in a burden on the cognitive controls. Therefore, reduced resources due to reduced activity of relevant areas may be the reason for the fMRI BOLD responses; nevertheless, the performance is better in the bimodal tasks. Efficient information processing on multiple perceptual tasks and its relationship to attentional modulation needs to be assessed in greater details in further studies. 
