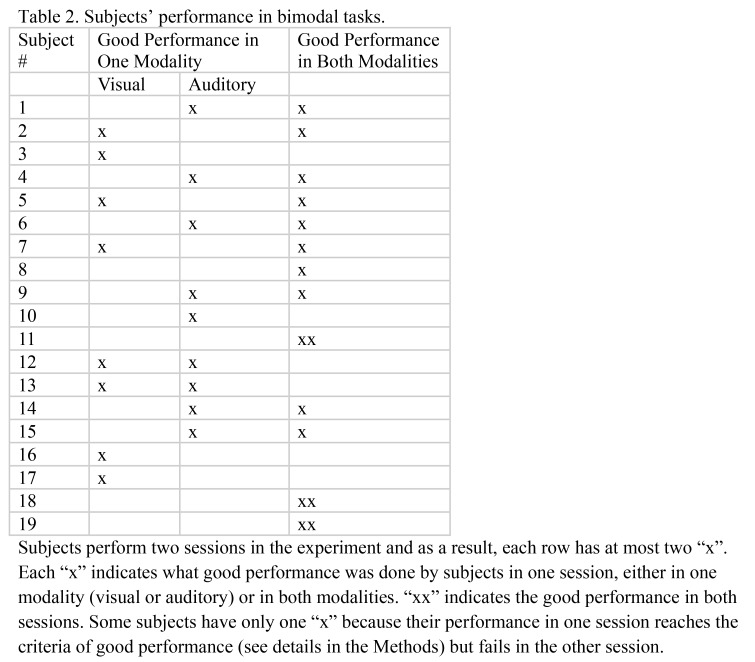# Correction: Brain Deactivation in the Outperformance in Bimodal Tasks: An fMRI Study

**DOI:** 10.1371/annotation/222f19cd-06e9-4f31-aec7-c8f0f185a8df

**Published:** 2014-01-03

**Authors:** Tzu-Ching Chiang, Keng-Chen Liang, Jyh-Horng Chen, Chao-Hsien Hsieh, Yun-An Huang

A formatting error was introduced during the production process for Table 2 in the PDF version only. Please see the corrected Table 2 and Table 2 notes here: 

**Figure:**